# Cattle Tick *Rhipicephalus microplus*-Host Interface: A Review of Resistant and Susceptible Host Responses

**DOI:** 10.3389/fcimb.2017.00506

**Published:** 2017-12-11

**Authors:** Ala E. Tabor, Abid Ali, Gauhar Rehman, Gustavo Rocha Garcia, Amanda Fonseca Zangirolamo, Thiago Malardo, Nicholas N. Jonsson

**Affiliations:** ^1^Centre for Animal Science, Queensland Alliance for Agriculture and Food Innovation, The University of Queensland, St. Lucia, QLD, Australia; ^2^Centre for Comparative Genomics, Murdoch University, Perth, WA, Australia; ^3^Department of Zoology, Abdul Wali Khan University Mardan, Mardan, Pakistan; ^4^Escola de Enfermagem de Ribeirão Preto, University of São Paulo, Ribeirão Preto, Brazil; ^5^Ribeirão Preto School of Medicine, University of São Paulo, Ribeirão Preto, Brazil; ^6^Institute of Biodiversity, Animal Health and Comparative Medicine, The University of Glasgow, Glasgow, United Kingdom

**Keywords:** ticks, immunity, tick resistance, tick susceptibility, cattle breeds, genetic variation, gene expression profiling, immune responses

## Abstract

Ticks are able to transmit tick-borne infectious agents to vertebrate hosts which cause major constraints to public and livestock health. The costs associated with mortality, relapse, treatments, and decreased production yields are economically significant. Ticks adapted to a hematophagous existence after the vertebrate hemostatic system evolved into a multi-layered defense system against foreign invasion (pathogens and ectoparasites), blood loss, and immune responses. Subsequently, ticks evolved by developing an ability to suppress the vertebrate host immune system with a devastating impact particularly for exotic and crossbred cattle. Host genetics defines the immune responsiveness against ticks and tick-borne pathogens. To gain an insight into the naturally acquired resistant and susceptible cattle breed against ticks, studies have been conducted comparing the incidence of tick infestation on bovine hosts from divergent genetic backgrounds. It is well-documented that purebred and crossbred *Bos taurus indicus* cattle are more resistant to ticks and tick-borne pathogens compared to purebred European *Bos taurus taurus* cattle. Genetic studies identifying Quantitative Trait Loci markers using microsatellites and SNPs have been inconsistent with very low percentages relating phenotypic variation with tick infestation. Several skin gene expression and immunological studies have been undertaken using different breeds, different samples (peripheral blood, skin with tick feeding), infestation protocols and geographic environments. Susceptible breeds were commonly found to be associated with the increased expression of toll like receptors, MHC Class II, calcium binding proteins, and complement factors with an increased presence of neutrophils in the skin following tick feeding. Resistant breeds had higher levels of T cells present in the skin prior to tick infestation and thus seem to respond to ticks more efficiently. The skin of resistant breeds also contained higher numbers of eosinophils, mast cells and basophils with up-regulated proteases, cathepsins, keratins, collagens and extracellular matrix proteins in response to feeding ticks. Here we review immunological and molecular determinants that explore the cattle tick *Rhipicephalus microplus*-host resistance phenomenon as well as contemplating new insights and future directions to study tick resistance and susceptibility, in order to facilitate interventions for tick control.

## Introduction

Vector-borne pathogens cause diseases with a great impact on public and veterinary health and have accounted for 22% of emerging infections between 1940 and 2004 (Jones et al., 2008). As obligate hematophagous arthropod pests of vertebrates, ticks pose serious threats to beef and dairy cattle producers. It has been estimated that 80% of the world's cattle population is at risk from tick and tick-borne diseases (TBDs) causing estimated annual losses of US$ 22–30 billion (Lew-Tabor and Rodriguez Valle, 2016). The negative impact of ticks on cattle production is due to the direct effects of feeding, such as weight loss and damage of leather, and indirect effects, such as the transmission of tick-borne pathogens. The resulting diseases can potentially cause major production losses in livestock, thereby reducing farm incomes, increasing costs to consumers, and threatening trade between regions and/or world markets.

Since the establishment of extensive vector control programs, a steady decline in vector-borne diseases was observed last century, however recently the emergence and re-emergence of vector-borne diseases has been observed. This re-emergence may be linked to new global trends associated with changes in animal husbandry, urbanization, animal transboundary transportation, and globalization (Ogden and Lindsay, 2016). In this scenario, various approaches for tick control are in practice around the world in accordance with local legislation, environmental conditions, price based selection, and geography. Acaricide (synthetic pesticides) application is the most common component of tick control strategies, however the use of acaricides impose numerous limitations including the selective pressure for the development of more resistant ticks, environmental contamination, drug residues in food products, the expense of developing new acaricides, and the difficulty of producing tick-resistant cattle while maintaining desirable production characteristics (Willadsen, 2004; Abbas et al., 2014). Anti-tick vaccines are a very promising alternative to acaricide usage, however are still insufficient to confer protection against multiple tick species in various geographical regions (de la Fuente and Contreras, 2015; de la Fuente et al., 2016; Schetters et al., 2016).

Anti-tick immunity has been described in guinea pigs, cattle and rabbits, and refers to the capacity of previously exposed hosts to interfere with tick feeding and reproductive fecundity (Nuttall, 1911; Trager, 1939; Hewetson, 1972). A reduction in tick weight, duration of attachment, number of ticks feeding, egg mass, and molting success are some of the parameters measured to determine host anti-tick immunity (Trager, 1939). For the first time, Nuttall (1911) demonstrated host immunity to ticks as a phenomenon of natural immunity in humans. Experimentally, acquired resistance to tick infestation was observed by Trager (1939), who noticed that after repeated infestation of *Dermacentor variabilis* on guinea pigs, the host developed resistance to subsequent tick infestation, shown by the decreasing number of successfully feeding larvae. Furthermore, it was found that as compared with larvae infesting a host with no previous exposure to ticks, larvae infesting resistant hosts weighed less. Several researchers continued to observe host resistance to tick feeding affecting each tick life stage (Gregson, 1941; Feldman-Muhsam, 1964; Wikel, 1996). Various immunological determinants have been examined that influence host resistance to tick infestation including a high level of eosinophils, basophils, T cells, mast cells, specific immunoglobulins, histamine, and changes to gene transcription profiles (Kemp and Bourne, 1980; de Castro and Newson, 1993; Kashino et al., 2005; Veríssimo et al., 2008; Kongsuwan et al., 2010; Piper et al., 2010; Engracia Filho et al., 2017).

Bovines present contrasting, heritable phenotypes for infestation with *Rhipicephalus microplus* and related tick species as a consequence of co-evolution of resistant cattle with ticks and also decades of selective breeding. The *R. microplus* tick has a strong preference for *Bos taurus taurus* cattle over highly resistant *Bos taurus indicus* cattle (Wambura et al., 1998; Porto Neto et al., 2011b; Jonsson et al., 2014; Biegelmeyer et al., 2015). In this article we review the tick:host physical interface, genetic and molecular studies, and immunological determinants of bovine host resistance to ticks.

## Tick-host physical interface

The cattle tick *R. microplus* co-evolved with Asian bovines (zebu breeds) and due to the global migration of *B. t. taurus* European breeds for dairy production during the eighteenth–nineteenth centuries, this tick spread across tropical and sub-tropical regions of the world (Frisch, 1999; Estrada-Peña et al., 2006; Barré and Uilenberg, 2010). Currently *R. microplus* is considered to be a species complex, in which there are recognized geographic differences between the 5 clades including 3 clades of *R. microplus* (A, B, and C), as well as *R. australis* and *R. annulatus* (Burger et al., 2014; Low et al., 2015). Each taxa transmits both anaplasmosis and babesiosis and each have a parasitic life cycle on cattle for ~21 days. They will be described collectively as *R. microplus* or simply as cattle ticks in this review. Cattle ticks are attracted to their hosts through stimuli such as carbon dioxide, temperature, vibrations, visual stimuli, and odor (Osterkamp et al., 1999). The susceptible European (*B. t. taurus*) breeds which were introduced into regions in which *R. microplus* is endemic failed to resist tick infestation to the same extent as tropical *B. t. indicus* breeds, which have developed an effective anti-tick immune response (Frisch, 1999).

The immune response varies among newly introduced European cattle (Taurine breeds, susceptible hosts) whereas Asian bovines (zebuine breeds or resistant hosts) co-evolved with ticks (Utech et al., 1978). Physical barriers that affect tick resistance include density of the fur coat, skin thickness, skin pigmentation (light or dark), skin vibration and/or self-cleaning ability, tongue papillae, and odor (de Castro et al., 1985; Spickett et al., 1989; Veríssimo et al., 2002, 2015; Martinez et al., 2006; Gasparin et al., 2007). In addition to physical differences between resistant and susceptible hosts, their behavior also affects the *R. microplus* parasitic load. Self-grooming is widely used by cattle as an important defense mechanism against ticks (Riek, 1956; Snowball, 1956; Bennett, 1969) and the level of resistance may possibly be associated with tongue morphology. For example the papillae from tick-resistant breeds have smaller spacing, which is more effective in removing *R. microplus* larvae from the skin (Veríssimo et al., 2015). However, there is also conjecture that resistant breeds simply groom more often (Kemp et al., 1976). It has been suggested that innate characteristics such as thinner coats and lower fur density have direct impacts in decreasing tick preferential attachment and infestation (Spickett et al., 1989; Veríssimo et al., 2002; Gasparin et al., 2007; Marufu et al., 2011). However, other studies have shown that skin features have no influence on tick infestation (Wagland, 1978; Doube and Wharton, 1980). Evidence for resistance of cattle to ticks due to physical parameters is scarce and further studies to examine the mechanisms that govern these physical phenomena are still needed.

## Immunological determinants associated with host resistance: host counter attack?

It is well-established that cattle have three subclasses of IgG (IgG1, IgG2, and IgG3), and during blood meals ticks ingest a substantial amount of IgG (Knight et al., 1988; Symons et al., 1989; Kacskovics and Butler, 1996; Rabbani et al., 1997; Gudderra et al., 2002; Saini et al., 2007). Host IgGs can be found in the tick hemolymph and are potentially biologically active against specific tick proteins (Ben-Yakir et al., 1987). Furthermore, specific host antibodies neutralize the tick salivary pharmacopeia and can damage the tick by binding to tick internal organs such as salivary glands, midgut, or ovaries (Ackerman et al., 1981; Willadsen and Kemp, 1988; Tellam et al., 1992). In other tick-host systems (*Dermacentor andersoni* and guinea pigs), antibodies have been shown to mediate inflammatory reactions by triggering effector-cell recruitment and cellular immune response as a consequence of both Fc receptor activation of leukocytes and complement activation that are harmful to the tick, also an immune mechanism in human auto-immune disease syndromes (Wikel and Whelen, 1986; Hogarth, 2002).

It was documented previously that the passive transfer of plasma from genetically immune resistant animals to naïve hosts, increases resistance to tick challenge and this response was believed to be mediated by antibodies (Roberts and Kerr, 1976; Shapiro et al., 1986). The pattern of antibody responses to immunogens from tick salivary glands and guts have been reviewed by different research groups (Willadsen, 1980; Wikel, 1982; Kaufman, 1989; Kashino et al., 2005; Cruz et al., 2008; Piper et al., 2009, 2010; Garcia et al., 2017).

Some studies have shown that during laboratory animal infestations, such as guinea pigs, rabbits and mice, reactive antibody titers to tick salivary antigens increased (Allen and Humphreys, 1979; Allen, 1989). The densities of *Amblyomma hebraeum, Rhipicephalus appendiculatus*, and *Rhipicephalus evertsi evertsi* ticks was higher on the susceptible breed (*B. t. taurus* Hereford) as compared to resistant cattle (*B. t. indicus* Brahman) with a positive correlation between the level of tick infestation and the level of IgG in susceptible hosts (Rechav, 1987). Piper and colleagues confirmed this correlation noting that susceptible cattle (Holstein-Friesian) have higher levels of tick specific IgGs compared to Brahmans suggesting that these antibodies do not confer immunity to ticks (Piper et al., 2008, 2009, 2010). As with other immune parameters in high and low resistance animals, the interpretation of data can be problematic as a susceptible animal will have more ticks feeding at any time, which would in turn be expected to result in a higher antigenic challenge. However, the negative relationship between IgG levels and host resistance was later confirmed and shown to be independent of the number of feeding ticks, using Santa Gertrudis cattle [a stable composite breed of *B. t. taurus* (5/8) and *B. t. indicus* (3/8)], in which there is wide variation in host resistance to ticks (Piper et al., 2017). Susceptible animals had significantly higher tick-specific IgG1 antibody titres (to several tick antigens including adult female salivary glands and guts, and whole larvae) compared to tick resistant cattle (Piper et al., 2017). In contrast, Kashino et al reported that tick saliva-specific IgG1 and IgG2 antibodies decreased in susceptible (Holstein) compared to resistant (Nelore) cattle where the IgG levels remained the same, however, only IgG levels to tick salivary antigens were examined (Kashino et al., 2005). Previous studies have surmised that there are genetic differences in the bovine host's ability to elicit antibody responses to antigens in *R. microplus* and *D. andersoni* tick saliva (Whelen et al., 1986; Opdebeeck and Daly, 1990). Despite the fact that differences in the IgG levels against tick antigens between heavy or light infestations have been reported, there is individual variation in the same bovine breed with respect to humoral immune responses to tick antigenic molecules (Cruz et al., 2008). In addition, despite most studies reporting increased total IgG production against wide ranging tick antigens in susceptible breeds compared to resistant, IgG responses to salivary proteins were significantly higher in tick naïve resistant hosts (Nelore) at the first larval challenge (Garcia et al., 2017).

Variation in IgG2 allotypes has been associated with variation in immune responses to pathogens. When two allotypes IgG2a and IgG2b were found to differ in sequence at the CH1–CH3 regions it was reported that IgG2b was more able to initiate the bovine complement cascade while animals with the IgG2a allotype were more susceptible to extracellular pyogenic pathogens (Heyermann and Butler, 1987; Bastida-Corcuera et al., 1999). Other studies have shown that the distribution and presence of IgG2 allotypes differed significantly between taurine and indicine breeds (Butler et al., 1994; Carvalho et al., 2011). Blakeslee et al. (1971) described that ~80% of susceptible bovines (Holstein) have the IgG2a allotype and that the IgGγ2b was rare in these animals (Blakeslee et al., 1971). Recently, it was shown that the IgGγ2a allotype was significantly more frequent in taurine hosts (tick susceptible) and IgGγ2b was significantly frequent in indicine cattle (tick resistant) (Carvalho et al., 2011). Male tick saliva contains IgG-binding proteins (IGBPs) secreted into the host which assists the female tick to evade the host immune response (Wang and Nuttall, 1999; Santos et al., 2004; Gong et al., 2014). Carvalho et al. (2011) suggested that certain IgG2 allotypes may hinder the function of these tick IGBPs.

Aside from IGBPs, other tick specific proteins have been examined in terms of their immune recognition by tick resistant and susceptible cattle. A *R. microplus* recombinant serine protease inhibitor (Serpin- rRMS-3) was recognized by resistant bovines and not susceptible, suggesting that RMS-3 could be a protective antigen (Rodriguez-Valle et al., 2012). Another study by the same group demonstrated that host responses to six *R. microplus* lipocalins (LRMs which include tick histamine binding proteins) were higher in resistant cattle (Rodriguez-Valle et al., 2013). Both RMS-3 and the LRM proteins were identified based on the *in silico* identification of B cell binding epitopes. In addition, predicted T cell epitopes from 3 LRMs stimulated the generation of a significantly higher number of interferon gamma (IFN-γ) secreting cells (consistent with a Th1 response) in tick-susceptible Holstein–Friesians compared with tick-resistant Brahman cattle. In contrast, expression of the Th2-associated cytokine interleukin-4 (IL4) was lower in Holstein–Friesian (susceptible) cattle when compared with Brahman (resistant) cattle (Rodriguez-Valle et al., 2013). IL4 is known to decrease the production of Th1 cells and IFN-γ, and is thus a key regulator of both the humoral and adaptive immune responses.

The immunological parameters of tick resistance have been shown to differ between tick susceptible and tick resistant breeds as well as within the same breeds. The studies reported differ in the parameters of trials and tick infestations, for example, the use of tick naïve cattle artificially infested or the use of cattle naturally exposed to ticks (Kashino et al., 2005; Piper et al., 2008, 2009). The study undertaken by Kashino et al. (2005) used susceptible cattle that had been treated with acaricides when high tick numbers were observed and the cattle had been vaccinated with GAVAC (Bm86 based tick vaccine), introducing additional variables to the study. Further studies examining specific tick proteins to compare divergent host immune responses are still warranted.

## Molecular genetic variants associated with host resistance

Numerous studies have attempted to identify genetic markers for host resistance to tick infestation and they are summarized and discussed by Porto Neto et al. (2011b) and Mapholi et al. (2014). Approaches have included immunological methods (Stear et al., 1984, 1989, 1990); protein-based analyses (Ashton et al., 1968; Carvalho et al., 2008); candidate gene sequence or genotype (Acosta-Rodriguez et al., 2005; Martinez et al., 2006; Untalan et al., 2007); genomic detection of quantitative trait loci (QTL) using SNPs or microsatellites, with or without fine mapping (Barendse, 2007; Gasparin et al., 2007; Regitano et al., 2008; Prayaga et al., 2009; Machado et al., 2010; Porto Neto et al., 2010a, 2011a; Turner et al., 2010; Cardoso et al., 2015; Mapholi et al., 2016; Sollero et al., 2017). There is one example of a meta-analysis of genomic association with transcriptome in tick infestation (Porto Neto et al., 2010b). Although this appears to represent a large body of science, it has generated relatively little data which can be used for improved genetic selection. It can be concluded from the studies on the major histocompatibility complex (MHC, also referred to as the bovine lymphocyte antigen (BoLA) system) that the MHC makes a contribution to variation in resistance however there is no single, consistent genotype of any gene in the MHC that is associated with high or low resistance to ticks across breeds and production systems. A number of QTL markers have been identified using microsatellites and SNPs, however these have mostly been inconsistent and the loci have had relatively weak effects. The research of Barendse (2007) and Turner et al. (2010) found several significant loci but most of them had effects in the order of 1% of the phenotypic variation in tick infestation. The lack of consistent and strong findings is not surprising. Counting ticks is difficult and time consuming thus studies resort to scoring systems, which are less precise than counts, and this can have an effect on heritability. Alternatively, the numbers used tend to be relatively small and studies are underpowered. The most robust report is that by Turner et al. (2010), who reported on a study in which ticks were counted and heritability was a respectable 37%, and which used 1,960 cattle. In contrast, Prayaga et al. (2009) used a scoring system, 900 animals and estimated heritability of tick score to be 9%. Furthermore, a study examining the genomic prediction for tick resistance in Braford (Brahman x Hereford/tick resistant x tick susceptible breed, respectively) and Hereford cattle in Brazil showed that genomic selection for tick resistant Braford cattle may be achievable (Cardoso et al., 2015). A recent trait tag-SNP approach by the same group reported 914 SNPs explaining more than 20% of the estimated genetic variance for tick resistance (Sollero et al., 2017).

Despite the challenges of the genomic approach to identifying either mechanisms or markers for host resistance to ticks in cattle, they have enabled the identification of allelic variation in genes that are very likely to influence the trait. The *ELTD1* gene (EGF, latrophilin, and seven transmembrane domain containing 1) was identified from GWAS in dairy and beef cattle (Prayaga et al., 2009; Turner et al., 2010). Its association with the host resistance phenotype was confirmed but its effect was limited to <1% of the total phenotypic variation in the trait (Porto Neto et al., 2011a). Similarly, haplotypes that included the *ITGA11* gene (integrin alpha 11) were significantly associated with tick burden and explained about 1.5% of the variation in the trait. Finally, the potential functional role of allelic variation in a gene identified by the same GWAS studies (Prayaga et al., 2009; Turner et al., 2010) *RIPK2* (serine-threonine kinase 2) was further examined using knock out mice (Porto Neto et al., 2012). This gene is known to play an essential role in the modulation of innate and adaptive immune responses and it was found that it influenced the recognition of tick salivary antigens by mice.

Limited association of tick burden or phenotype to the genotype is currently available and large bovine genomic meta-analyses may contribute to the identification of within breed markers for tick resistance in the future.

## Variation in gene expression among resistant and susceptible hosts and relationship with immune responses

Bioactive molecules secreted by *R. microplus* ticks into the skin of the host during attachment and blood feeding stimulate host effective responses. The variation in the mechanisms by which each host breed responds to each of these bioactive molecules likely results in different levels of resistance. It is well-established that the resistance to tick infestation is due to a complex set of responses, however the specific mechanisms and their relative importance continues to be the subject of debate.

**Figure 1:**
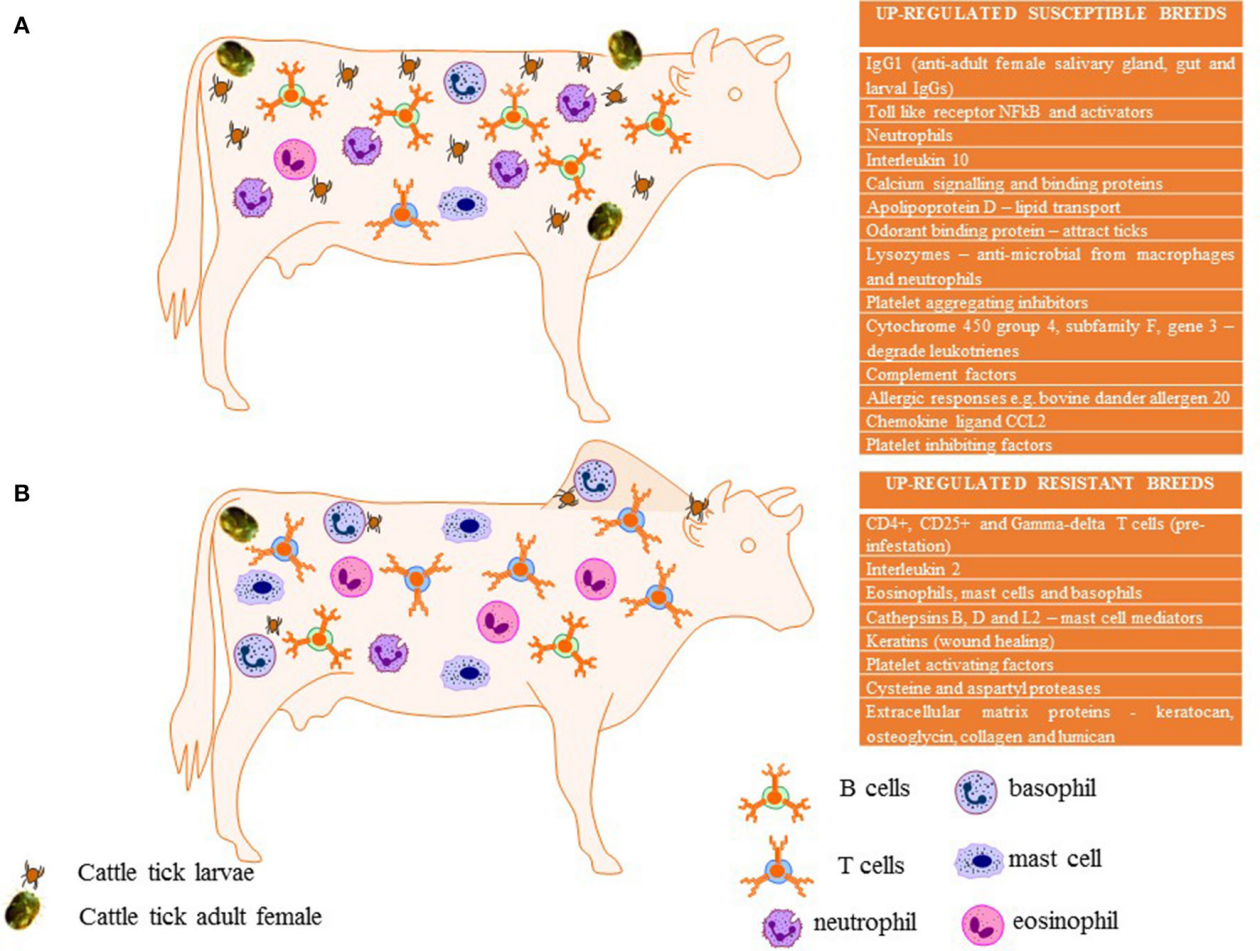
Summary of expression and immunological profiles *commonly* associated in tick susceptible **(A)** compared to tick resistant breeds **(B)** of cattle as identified in Table 1. This diagram was created using images from Motifolio Inc.

Table 1 summarizes selected up-regulated genes including those that are potentially associated with immune responsiveness, blood coagulation, calcium regulation, and/or wound healing from several studies undertaken to date. The parameters of all of the studies differ from each other including: the number of biological replicates, the number of larvae used in infestations, the breeds and subspecies used, their prior exposure, the methodology and platform used to measure host responses (immunohistochemistry, microarray platforms, qPCR), the timing of sample collection, and the samples analyzed (skin or blood). Without undertaking a formal meta-analysis of the original data, we have attempted to summarize differences and similarities in relation to susceptible vs. resistant animals among reports. The similarities identified are presented diagrammatically in Figure 1 with text listing commonly up-regulated cells or genes in susceptible (Figure 1A) and resistant (Figure 1B) cattle.

**Table 1:** Summary of differentiating characteristics of tick susceptible vs. tick resistant cattle (bold font indicates correlation between studies of certain transcripts and or other markers—genetic, cellular, immunohistochemistry) in response to *Rhipicephalus microplus* ticks.

**Methodology (cattle breeds compared)**	**Susceptible**	**Resistant**	**Main conclusion and reference**
Cellular IgG isotyping; Cattle naturally infested on pasture; susceptible cattle treated with acaricide when tick counts were >500. Cattle vaccinated with GAVAC. (Holstein and Aberdeen *B. t. taurus* vs. Nelore *B. t. indicus*)	IgG1 and IgG2 decrease (anti-salivary gland antibodies only)	IgG1 and IgG2 no change	IgG suppression by tick infestation on susceptible cattle. Kashino et al., 2005
Genetics study, cattle naturally infested in pasture and at 10–14 months infested with 10,000 tick larvae, PCR to determine allele frequency (F2 population of composites *B. t. indicus* Gyr 1/2 x *B. t. taurus* Holstein 1/2)		**BoLA Class II** DRB3.2	The MHC Class II DRB3.2 allele is associated with tick resistance. Martinez et al., 2006
Microarray gene expression, natural tick infestation for ~6 months (“naïve” sample taken post field infest), followed by 5 artificial tick challenges over 17 months with 20,000–25,000 larvae (Hereford Shorthorn cattle- tropically adapted *B. t. taurus*)	**B-cell CLL/lymphoma 10** **Collagen**, pro α **Apolipoprotein** **Keratin** **Immunoglobulins** IL13 receptor α 1 precursor	**Cathepsin** B **Collagens** (type I α2; type III pro; type VI, α 3) **Complement** component 1, q subcomponent, α	Susceptible cattle upregulated immune responsive genes and those involved in protein synthesis. **BDA20, Odorant Binding Protein (OBP)** and the dendritic cell protein-B5 over expressed by both groups. Wang Y. H. et al., 2007
Genetic microsatellite and cytokine qPCR of 4 breeds, 17 months natural tick infestation (*B. t. indicus* Nelore, Composite Canchim 5/8 *B.taurus* 3/8 *B. t. indicus* × Nelore, *B. t. taurus* Aberdeen Angus × Nelore, and *B. t. taurus* Simmental x Nelore)	IL4	**IL8** and **IL2** down-regulated	Tick counts were associated with IL4, IL2 is down-regulated in 3 genetically different groups of infested bovines compared to pre-infestation, and IL8 was down-regulated in resistant bovines compared to susceptible animals Regitano et al., 2008
RT-PCR gene expression of peripheral white blood cells, previous natural exposure to ticks, 7 weekly artificial infestations of 10,000 larvae while held in tick infested pasture. (Holstein Friesian *B.taurus* vs. Brahman cattle *B. t. indicus*)	Toll-like receptor pathway (TLR5, TLR7, TLR9, **NFKBp50**, MyD88, Traf-6, CD14, IL-1β) Chemokine ligands and receptors (**CCL2**, CCL26; CCR-1) **IL-10** **bovine dander allergen 20 (BDA20)**		15 transcripts increased significantly in the susceptible breed to suggest innate inflammatory processes Piper et al., 2008
Cellular, RT-PCR and microarray gene expression (microarray) of leukocytes– infestation as described above (Piper et al., 2008) (Holstein Friesian *B. t. taurus* vs. Brahman cattle *B. t. indicus*)	Higher WBC counts Low hemoglobin CD14+ and **MHC Class II** (macrophage cells) High tick specific immunoglobulins (larvae, adult female salivary gland and gut)—high IgG1 **CXCL10**	**CD4**+**, CD25**+ and γδ **T cells** **IL2**, IL2Rα, IL4Rα, TNFα, CCR-1, CCR-7, CXCL4, CD28, CD3E CD40 ligand	Susceptible cattle produce an innate inflammatory type response with high IgG1 titers suggesting also a T cell response. Resistant cattle produce a stable T cell mediated response. Piper et al., 2009
RT-PCR gene expression of calcium signaling genes in skin biopsies. Natural field exposure to ticks, followed by treatment and at 8 months artificial infestation of 10,000 larvae. Skin biopsies at 0, 3, and 24h post infestation (Belmont Red—composite breed ~ 1/2 *B. t. taurus* and 1/2 *B. t. indicus*, tropically adapted)		**Calcium signaling genes** (significant at 24 h post-exposure): *AHNAK* nucleoprotein (desmoyokin) *CASQ* (Calsequestrin) **IL2** (Interleukin-2) NFAT2CIP (nuclear factor of activated T-cells, calcineurin-dependent) PLCG1 (phospholipase C, γ1)	The significant elevation of some calcium dependent genes following tick exposure suggests that the calcium pathway might be responsive to parasite exposure and could contribute to host immune response. Bagnall et al., 2009
Protein and mRNA expression- infestation as described above (Bagnall et al., 2009) of skin biopsies at 0, 3 and 24 h post-infestation. (Belmont Red – composite breed ~ 1/2 *B. t. taurus* and 1/2 *B. t. indicus*, tropically adapted)		**Keratins** lipocalin 9 epidermal barrier catalyzing enzyme transglutaminase 1 transcriptional regulator B lymphocyte-induced maturation protein 1 lipid processing proteins	Resistant cattle have physically stronger epidermal layers of the skin Kongsuwan et al., 2010
Host gene expression microarrays (skin); infestation as described above Piper et al., 2008 (Holstein Friesian *B. t. taurus* vs. Brahman *B. t. indicus* cattle)	**Chemokine ligands** (C-X-C motif) 2 and 5; (C-C motif) 2 and 8 – CXCL2, CXCL5, **CCL2**, CCL8 **Complement** component 1 and 3; Complement factor D (adipsin) **IL8** **Major histocompatibility complex Class II DRB3** Chemokine Regakine 1 (chemotactic activity for neutrophils) Spleen trypsin inhibitor; Plasminogen activator, tissue; Serpin peptidase inhibitor, clade F; Proapoptotic caspase adapter protein (inflammatory role, recruitment of immune cells to infected tissue); Peptidoglycan recognition protein 1 (inducer of TNFα and IFN γ); **Apolipoprotein D** **Lysozyme** Prostaglandin D2 synthase (inhibitor of platelet aggregation)	Keratocan, osteoglycin, lumican, **Collagen**, (types I and III); Lysyl oxidase-like 4 (formation of collagen) Down-regulated **Cytochrome P450** (**CYP4F3**, CYP11A1) Serpin peptidase inhibitor, clade A (alpha-1 antiproteinase, antitrypsin); Phospholipase A2, group VII (platelet-activating factor acetylhydrolase, plasma); Secreted phosphoprotein 2 (cystatin); Procollagen C-endopeptidase enhancer Lipid metabolism Amino acid metabolism Oxidoreductases	Genes involved with inflammatory processes and immune responsiveness upregulated in susceptible cattle. Genes encoding consistuents of the extracellular matrix were up-regulated in resistant cattle. Piper et al., 2010
qPCR analysis after artificial tick infestation, skin biopsies from tick lesions. (Gyr -*B. t. indicus* × Holstein - *B. t. taurus*)	**Calcium-dependent signal transduction** (**S100A7)**, histamine releasing protein (TPT1), epithelial calcium channel 2 (TRPV6) cysteine proteinase inhibitor (CST6)		Susceptible cattle develop hypersensitive reaction which is not protective Nascimento et al., 2010
EST analysis, natural tick infestation followed by acaricide treatment and artificial infestation of 10,000 larvae, biopsies collected at Days 5 and 12 from base of tail (F2 population from Gyr -*B. t. indicus* × Holstein - *B. t. taurus*)	CD44 antigen (lymphocyte activation) CD63 antigen (marker for activated basophils and IgE-mediated allergy) ADAM metallopeptidase **odorant binding protein** poly A binding protein ribosomal proteins	MHC Class antigen 1 **Cathepsin** L2 precursor **Collagen** (type I alpha; type III alpha) **Keratins** Ribosomal proteins	Structural proteins and MHC Class I in resistant cattle; Immune response transcripts in susceptible cattle Nascimento et al., 2011
Immunohistochemistry, infestation of Holstein Friesian and Brahmans as described by (Piper et al., 2008) above. Infestation of tick naïve Santa Gertrudis cattle−10,000 larvae artificially weekly for 13 weeks followed by natural infestation on pasture (Holstein Friesian *B. t. taurus* vs. Brahman *B. t. indicus*) (Composite Santa Gertrudis breed *B. t. taurus* 5/8 × *B. t. indicus* 3/8)	CD45, CD45RO (Holstein-Friesian) Mixed CD45 and CD45RO reactions (Santa Gertrudis)	**CD4**+, CD8+, **CD25**+ and γδ **T cells** **MHC Class II cells** Mixed CD45 and CD45RO reactions (Santa Gertrudis)	Higher number of γδ T cells present in the skin of tick naïve resistant cattle. Epitopes recognized by some antibodies might not be present on the cell populations from all breeds of cattle or might be expressed in different levels. Santa Gertrudis resistance associated with lower cellular reaction at the larval attachment sites. Constantinoiu et al., 2010, 2013; Jonsson et al., 2014
Microarray gene expression (skin), natural infestation on pasture (10 months), acaricide treatment, followed by artificial infestation 10,000 larvae, skin biopsies 0, 24 and 48 h post-artificial infestation. (F2 of composite Gyr -*B. t. indicus* × Holstein - *B. t. taurus*)	**Calcium ion control genes** **Cytochrome P450** (CYP4F3)—**leukotriene** (allergy inducing chemical mediator) degradation	**Complement factors** 1, 2 and 3 complexes (C1, C2, C3); complement factor 4 binding protein; complement factor B properdin, galectin-1 Coagulation Factor XIIIa Lipid metabolism	Lipid metabolism in inflammation control in resistant cattle. Acute phase response impaired in susceptible cattle. Carvalho et al., 2014
RT-PCR gene expression of peripheral white blood cells, natural tick infestation followed by acaricide treatment, subsequent artificial infestation with 20,000 larvae, blood samples taken at 0, 24, and 48 h post-infestation. (F2 of composite Gyr -*B. t. indicus* × Holstein - *B. t. taurus*)	**CXCL10** and CXCL8 were down-regulated	**CD4**+**, CD25**+ and γδ **T cells** **IL10**, FoxP3, **CXCL10**, CD25	A correlation between T γδ cell activity and immunological mechanisms in resistant cattle Domingues et al., 2014
Cellular and humoral responses (blood), 10,000 larvae artificially weekly for 13 weeks followed by natural infestation on pasture, blood collected 21 days post 1st infestation and weekly to measure changes in measure parameters (Composite Santa Gertrudis breed *B. t. taurus* 5/8 x *B. t. indicus* 3/8)	High tick-specific (anti-salivary and gut, anti-larval) **IgG1 titers**; variable IgG2 responses Decreased hemoglobin, decreased RBC count Increased **CD25**+, **CD4**+, WC1+	No IgG2 Increased **CD4**+ **cells** (prior to infestation) Decreased CD3+, **CD4**+, CD8+ Increased **MHCII**+, WC3+ and CD14+ cells	A non-protective high level of IgG1 in tick susceptible cattle. Piper et al., 2017
Microarray gene expression (skin) and histology Acaricide treatment of calves prior to artificial tick infestation with 10,000, skin biopsies with and without feeding ticks were taken prior to infestation (day 0) followed by 2 days (larvae) and 9 days (nymphs). Biopsies without feeding ticks to emulate “stressed” phenotype. Selected immune response genes differentially expressed (>2-fold) by larvae in comparison to baseline and stress responses (no ticks) in susceptible and resistant cattle (Table S5). (Holstein *B. t. taurus* vs. Nelore *B. t. indicus*)	CD209 antigen (leads to **IL10** production) Fibroleukin (fibrinogen type 2) Secretogranin-2 Secretoneurin (chemotaxis of monocytes and eosinophils) CXC6 (downregulated); CCL3, CCL4 high affinity **immunoglobulin** epsilon receptor subunit alpha precursor (**NF-kB**, MAPK activation) **Complement factor** D **Complement** C1q tumor necrosis factor-related protein 7 precursor T-cell surface glycoprotein CD3 delta chain platelet-derived growth factor receptor-like protein precursor (wound healing) Serine/threonine-protein kinase 1 Secreted phosphoprotein 24 (endopeptidase, platelet degranulation) **Keratin** **Protein S100**-G (calcium binding) **Lysozyme**	CD3+ and γδ **T cells** WC1+ Activation of basophils interferon-induced GTP-binding protein Mx2 Interferon alpha-inducible proteins 6 and 27 IL3 **cathepsin** D precursor	Susceptible cattle produce more tick attracting volatiles. Resistant hosts expose ticks to an earlier inflammatory response. Franzin et al., 2017

Gene expression studies have been undertaken using peripheral blood leukocytes (PBL) and skin tick bite sites. These studies have included host qPCR, EST libraries, microarray analyses and cDNA library/next generation sequencing. The findings are summarized in Table 1.

Studies on PBL suggest that resistant hosts are more likely to develop a stable T-cell-mediated response against *R. microplus*, while susceptible cattle demonstrated cellular and gene expression profiles consistent with innate and inflammatory responses to tick infestation (Kashino et al., 2005; Piper et al., 2009). The up-regulation of genes in tick susceptible cattle involved in inflammatory and other important immunological responses mediate a greater natural potential to develop higher pro-inflammatory responses in comparison to tick resistant animals.

Gene expression studies on skin taken from larval attachment sites have demonstrated that cytokines, chemokines, and complement factors were differentially expressed between naïve-skin and infested skin in susceptible Holstein–Friesian cattle. It was also found that immunoglobulin transcripts were differentially expressed in infested skin from Holstein-Friesian compared to resistant Brahman cattle. Therefore, the chronic pathology established in *B. t. taurus* cattle might facilitate the tick feeding process (Piper et al., 2010). In addition, extracellular matrix genes such as: keratocan, osteoglycin, collagen, and lumican were up-regulated in infested-skin from *B. t. indicus* resistant Brahman cattle. In a study involving coagulation in skin from resistant and susceptible cattle infested with *R. microplus* (Carvalho et al., 2010b), susceptible hosts had an increased blood clotting time at tick hemorrhagic feeding pools in comparison to normal skin and the skin of resistant hosts. Furthermore, the host resistant phenotype affects the transcript of genes associated with anti-hemostatic proteins in the salivary glands of *R. microplus*, with transcripts coding for anti-coagulant proteins expressed at a higher level in ticks fed on susceptible hosts compared to ticks fed on resistant hosts (Carvalho et al., 2010b).

In the same experiment where PBL gene expression was studied in infested indicine and taurine cattle, the authors examined the response to infestation and larval attachment in bovine skin (Piper et al., 2010). The susceptible cattle displayed an intense cellular inflammatory response at the tick attachment site, i.e., genes involved in inflammatory processes and immune responses including those which encode for matrix proteins were up-regulated in tick-infested susceptible cattle, but not in resistant hosts. Nascimento et al. (2010) constructed cDNA libraries from skin biopsies from resistant and susceptible cattle to evaluate the pattern of gene expression of three calcium-binding-proteins. The results showed that genes coding for translationally controlled tumor protein (*1-TPT1*), calcium channel protein transient receptor potential vanilloid 6 (*TRPV6*) and cysteine proteinase inhibitor (*CST6*) were highly expressed in susceptible cattle compared to resistant cattle (Nascimento et al., 2010). Also, a microarray study using samples from tick infested cattle to evaluate the profile of gene expression during *R. microplus* larvae attachment showed differentially expressed genes involved in lipid metabolism, inflammation control and impairment of tick infestation in resistant cattle (*B. t. indicus* Nelore) (Carvalho et al., 2014). Conversely, in susceptible cattle (*B. t. taurus* Holstein) the acute phase response appeared impaired but this study confirmed the up-regulation of calcium ion control genes which correlates with the calcium binding proteins reported by Nascimento et al. (2010). Franzin et al. (2017) also report the up-regulation of protein S100G, another calcium binding protein, in susceptible cattle. An earlier qPCR study showed higher up-regulation of calcium signaling genes in a tick resistant composite breed in response to ticks most predominantly at 24 h post-infestation with larvae (Belmont Red) (Bagnall et al., 2009). Calcium signaling, calcium binding and/or calcium ion control genes and their functions in tick resistant and tick susceptible cattle warrant further specific examination.

Another host gene expression study was reported recently demonstrating that resistant cattle (*B. t. indicus* Nelore breed) up-regulated the expression of fewer genes encoding enzymes producing volatile compounds that render them less “attractive” to ticks compared with susceptible cattle (*B. t. taurus* Holstein breed) (Franzin et al., 2017). This finding is consistent with the theory associated with odor (Osterkamp et al., 1999) described above. The study also reported that resistant hosts when exposed to ticks mount an earlier inflammatory response than susceptible cattle (gene expression studies undertaken at 2 days post larval infestation using tick naïve cattle) (Franzin et al., 2017) which appears to disappear later (feeding nymphs at 9 days) but lingers in susceptible cattle.

Franzin et al. (2017) identified numerous novel immune response genes that were up-regulated in susceptible Holstein cattle including *FCER1A* the high affinity immunoglobulin epsilon receptor subunit alpha precursor which is known to be responsible for initiating an allergic response. The up-regulation of complement *C1QTNF7* (C1q tumor necrosis factor-related protein 7 precursor), an inducer of pro-inflammatory activators (Kishore et al., 2004) also concurs with the conclusions of tick susceptible cattle responding in a pro-inflammatory manner (Piper et al., 2010). Piper et al. (2008) had identified the up-regulation of Toll-like receptors (i.e., *NFkB*, nuclear factor kappa-light-chain-enhancer of activated B cells) that correlates with the activity of the complement C1Q identified by Franzin et al. (2017). In addition, *BCL10* (B-cell CLL/lymphoma 10) was up-regulated in susceptible cattle (Franzin et al., 2017) and is also known to activate *NFkB* (Wang M. et al., 2007). In addition fibroleukin is known to have a dual prothrombinase and immunoregulatory activity and was up-regulated in tick naïve (tick susceptible) cattle by larvae (Franzin et al., 2017). Immunoregulatory activity includes the suppression of T cell proliferation and cytokine production, mainly of Th1 and Th17 cells but not Th2 cells (Bézie et al., 2015), which is in contrast to other observations above suggesting that susceptible cattle mount a Th1 response. Functionally, Bézie et al. (2015) found that fibroleukin induced long-term allograft survival in a rat model through regulatory B cells which in turn suppress the proliferation of CD4+T cells. These cells are up-regulated in tick resistant cattle in the majority of studies undertaken. To summarize the results presented by Franzin et al. (2017), early responses to larvae (48 h post-infestation by naïve tick susceptible cattle) appear to show a mixed Th1 and Th2 response with tick susceptibility being associated with a Th1 response.

Several differences are noted among the comparisons of gene expression studies and as noted by Regitano et al. (2008) we generally concur that “*differences in gene expression of resistant cows compared to susceptible cows were breed-specific*.” However, there are some consistencies identified in the above studies. The presence of high densities of CD4+, CD25+, and γδ T cells are seen relatively consistently in resistant indicine cattle (see Table 1). The up-regulation of keratins and collagens is also common in resistant indicine cattle, with some divergent upregulation in susceptible breeds in fewer studies. The up-regulation of IgGs in susceptible cattle was reported by most researchers. MHC Class II and calcium binding proteins seem to be mostly up-regulated in susceptible breeds, with the latter commonly associated with susceptible breeds studied in Brazil. The expression of chemokine ligands varied greatly between studies and breeds with no identifiable consistency. Other genes that were up-regulated consistently in susceptible cattle include: apolipoproteins (lipid transport), lysozymes (anti-microbial, also found in macrophages and polymorphonuclear neutrophils), Toll like receptors, *NFkB* and NFkB activators *C1QTNF7* (complement C1q tumor necrosis factor –related protein 7) and *BCL10* (B-cell CLL/lymphoma 10) and several complement factors. IL10 is considered an anti-inflammatory cytokine perhaps induced early in susceptible cattle but IL10 appears to be associated with long term resistance in *B. t. indicus* Gyr cattle in Brazil (Domingues et al., 2014; Franzin et al., 2017). Cytochrome P450 enzymes are a superfamily of hemoproteins known to be involved in the synthesis or metabolism of various molecules and chemicals within cells. A P450 gene called CYP4F3 (cytochrome 450 group 4, subfamily F, gene 3) is known to degrade leukotrienes which are the chemical mediators of allergic responses (Karasuyama et al., 2011). CYP4F3 was downregulated in the skin of tick exposed resistant cattle (Brahmans, Piper et al., 2010) yet up-regulated in the skin of susceptible cattle (Holsteins, Carvalho et al., 2014) thus showing a correlation for the activity of this enzyme in tick susceptible cattle. A summary of all of these factors is presented in Figure 1.

Differential expression of genes coding for other host modifying enzymes were associated with resistant and susceptible phenotypes (Table 1). Although not identified in more than one study, the up-regulation of these factors appears to correlate to the relevant phenotype and are thus worthy of further description. For example the following were identified in different studies as up-regulated in tick resistant breeds: Cathepsin B (Wang Y. H. et al., 2007), Cathepsin L2 precursor (cysteine proteases, mast cell mediators) (Nascimento et al., 2011), Cathepsin D (aspartyl protease, mast cell mediator) (Franzin et al., 2017), serine peptidase inhibitor clade A (inhibits neutrophil elastase), phospholipase A2, group VII (platelet activating factor) (Piper et al., 2010), coagulation factors, and procollagen C-endopeptidase enhancer (metalloprotease inhibitor) (Piper et al., 2010); and conversely in tick susceptible breeds: serine peptidase inhibitor clade F (negative regulation of inflammatory response), spleen trypsin inhibitor, plasminogen activator (serine protease which produces plasmin which catalyzes the degradation of fibrin polymers in blood clots), prostaglandin D2 synthase (platelet aggregation inhibitor) (Piper et al., 2010), and phosphoprotein 24 (endopeptidase associated with platelet degranulation) (Franzin et al., 2017) were up-regulated.

Comparative transcriptomic studies of different life stages (larvae and adult females) have shown that ticks respond differentially according to whether they are sensing or feeding on a tick-susceptible or tick-resistant breed of cattle. A microarray study based on a *R. microplus* EST database (Wang M. et al., 2007), using sensing larvae (not attached to the host but contained within a fabric bag and able to sense the host) and feeding, adult females, which were collected from naïve, tick-susceptible Holstein Friesian *B. t. taurus*, and tick-resistant Brahman *B. t. indicus* cattle has been reported (Rodriguez-Valle et al., 2010). Ticks that were feeding on resistant cattle demonstrated the up-regulation of serpin 2, lipocalins and histamine binding proteins. A recent study utilized next generation sequencing to compare tick expression differences from larvae, nymph salivary glands and larval offspring of females - all fed on tick-resistant Nelore *B. t. indicus* and tick-susceptible Holstein *B. t. taurus* (Franzin et al., 2017). That study showed an increased number of transcripts that included evasins, immunosuppressant proteins, lipocalins (including histamine, serotonin, and odorant binding proteins), and reprolysin metalloproteases from ticks associated with susceptible cattle, and an increased number of chitinases and cysteine proteases from ticks associated with resistant cattle. The analysis also included larvae exposed to volatile compounds prepared from the same breeds of cattle and showed that resistant breeds produce less attractive volatiles (Franzin et al., 2017). The latter was thought to be correlated to the fact that ticks on susceptible cattle up-regulated odorant binding proteins. Not enough studies have been undertaken to draw any similarities between the molecular profiles of ticks from susceptible vs. resistant hosts particularly when different stages and breeds are compared. It is clear however that the tick gene expression profiles associated with tick-resistant vs. tick-susceptible cattle appear to be divergent.

Acquired immunity to tick infestation is established after a period of susceptibility to a primary infestation (Wagland, 1978). As confirmed above, gene expression profiles from tick resistant breeds appear to be congruent with a T-cell mediated response, while susceptible cattle exhibit innate and inflammatory responses with higher levels of tick specific IgG1. One quite consistent fact is that resistant cattle appear to be primed to respond to ticks with a higher presence of γδ T cells in the skin of tick naïve resistant cattle in comparison to susceptible. A formal meta-analysis of all gene expression studies where the data are in the public domain is theoretically possible but would likely be compromised by variation in the conditions of each of the studies. Each gene expression comparison study was undertaken using quite different conditions. The variable factors include: environment, season, naïve vs. tick exposed cattle, the use of acaricides post exposure prior to artificial infestations, infestation protocols including frequency and numbers of larvae, and comparative breeds including within breed studies.

## Cellular physiology associated with host resistance

Granulocytes (or polymorphonuclear leukocytes) are white blood cells characterized by the presence of granules in their cytoplasm and which perform different immune functions. They include neutrophils (most abundant), eosinophils, mast cells, and basophils. The inflammatory profile of the host skin contributes to resistance or susceptibility to tick infestation. Marufu et al. (2013) showed that tick susceptibility (*B. t. taurus* Bonsmara cattle) is associated with an immediate type hypersensitivity reaction. On the other hand, the resistance phenotype was linked to a delayed hypersensitivity reaction in *B. t. indicus* Nguni breed (Marufu et al., 2013) confirming the observations of Constantinoiu et al. (2010) with *B.t.taurus* Holstein Friesian (susceptible) and *B.t.indicus* Brahman (resistant) cattle. Table 2 summarizes the cellular profiles obtained in response to ticks with differences associated with: the tick stage (larvae, nymph and adults), use of previously tick-exposed or naïve cattle, timing of sample collection post-infestation, blood or skin samples, and methodologies used.

**Table 2:** Summary of inflammatory profile between susceptible and resistant breeds of cattle.

**Methodology (cattle breeds compared)**	**Susceptible**	**Resistant**	**Main conclusion and reference**
Histology of skin using larvae—several different breeds (*B. t. taurus* vs. *B. t. indicus*)	Higher **eosinophils** during secondary infestation		Wide variations in the magnitude of the lesions between different hosts Tatchell and Moorhouse, 1968
Skin histology at larval tick lesions, 3 h feeding on previously tick exposed cattle (tropically adapted Illawarra Shorthorn *B. t. taurus* susceptible vs. resistant)		Higher **mast cells** Higher **eosinophils** Higher **neutrophils**	Degree of mast cell disruption, eosinophil concentration and degranulation and the extent of epidermal vesiculation were all significantly higher in highly resistant hosts. Schleger et al., 1976
Skin biopsies from tick lesions (Gyr -*B. t. indicus* vs Holstein - *B. t. taurus*)	Lower mast cell counts	Higher **mast cell** counts	Mast cells important in host resistance Moraes et al., 1992
Dermal (upper and deep) mast cell counts (Nelore and Gyr -*B. t. indicus* vs. Holstein, Brown-Swiss and Jersey - *B. t. taurus*)	Holsten and Brown Swiss similar to Gyr Lowest mast cell counts – Brown-Swiss	High **mast cells** cell counts– Nelore Gyr - Similar to Holstein and Brown Swiss	Negative correlation between the number of mast cells in the upper dermis and tick counts. Mast cells important in tick resistance. Veríssimo et al., 2008
Immuno staining of skin sections (Brahman *B. t. indicus* vs. Holstein-Friesian *B. t. taurus*)	Higher **neutrophils**	Delayed hypersensitivity	γδ T cells might have a role in limiting the inflammatory process and preservation of the skin homeostasis in *B. t. indicus* cattle. Constantinoiu et al., 2010
In vitro binding of leukocytes and skin histology (Nelore -*B. t. indicus* vs. Holstein - *B. t. taurus*)	Adhesion molecules: leukocyte adhesion glycoprotein 1	Higher **basophils** Higher **eosinophils** Adhesion molecules: E-selectin (promotes adhesion of memory T cells)	Resistant cattle had significantly higher counts of basophils and eosinophils compared to susceptible breeds. Adhesion molecules indicate differences in resistant and susceptible hosts. Carvalho et al., 2010a
Cutaneous hypersensitivity responses to tick larval antigens in previously exposed cattle (Bonsmara *B. t. taurus* vs. Nguni *B. t. indicus*)	Intense cutaneous hypersensitivity response	Delayed hypersensitivity response	Marufu et al., 2013
Cellular responses to adult *R. microplus* in skin biopsies (Bonsmara *B. t. taurus* vs. Nguni *B. t. indicus*)	Higher basophils (lower than resistant cattle) Higher mast cells (lower than resistant cattle) Higher mononuclear cells (lower than resistant cattle) Higher **neutrophils** Higher **eosinophils**	Higher **basophils** Higher **mast cells** Higher mononuclear cells Higher neutrophils (lower than susceptible cattle) Higher eosinophils (lower than susceptible cattle)	Cellular responses showed higher counts of basophils, mast and mononuclear cells and lower neutrophil and eosinophil counts in resistant breeds. Marufu et al., 2014
Histopathology larvae and nymph with naïve cattle. (Holstein *B. t. taurus* vs. Nelore *B. t. indicus*)	Higher **eosinophils** (larvae) Higher **mast** cells (nymph, slightly higher compared to larvae bite site)	Higher **mast** cells (larvae), mast cells degranulated and decreased (nymph) Higher **eosinophils** (nymph) Higher **basophils** (nymphs)	Resistant hosts expose ticks to an earlier inflammatory response which is delayed in susceptible breeds. Franzin et al., 2017

*Bold fonts highlight common trends across different publications*.

Neutrophils are usually found in the blood stream and are the most abundant phagocyte. During host infection, neutrophils are quickly recruited to the site of infection i.e., skin in response to tick infestation. Neutrophils may favor infestation by destroying the extracellular matrix around the attached tick and thereby allowing access to blood for feeding (Tatchell and Bennett, 1969; Tatchell and Moorhouse, 1970). Similar numbers of neutrophils were found to be recruited in Holstein (susceptible, *B. t. taurus*) and Nelore cattle (resistant, *B. t. indicus*) (Carvalho et al., 2010a), with slightly higher neutrophils in the skin of susceptible cattle at early stages of infestation using naïve cattle of the same breeds (Franzin et al., 2017). Marufu et al. (2014) showed higher counts of neutrophils at the attachment sites of *R. microplus* in both resistant and susceptible breeds compared to non-infested skin, with higher counts also found in tick susceptible Bonsmara cattle compared to tick resistant Nguni cattle (Marufu et al., 2014). Higher levels of neutrophils do not seem to show a protective role against *R*. *microplus* infestation and feeding larvae demonstrated a high ingestion of neutrophils in susceptible *B. t. taurus* Holstein Friesian cattle (Constantinoiu et al., 2010). Moreover, activated neutrophils lead to a calcium ion influx which could correlate with the common up-regulation of calcium binding proteins in susceptible cattle gene expression studies (Table 1). Overall, higher neutrophil densities in the skin at the site of a tick attachment appear to be associated with the tick susceptible phenotype.

Eosinophils have long been known to be associated with parasite infections and allergy, with several immune functions having been only recently elucidated. For example, recent evidence suggests that eosinophils suppress Th17 and Th1 responses via dendritic cell regulation and also activate basophil degranulation (Wen and Rothenberg, 2016). Eosinophils may influence the tick resistant phenotype due to their role in the translocation of mast cell histamine and lysosomal enzymes to the feeding site lesion, and by impairing tick attachment (Schleger et al., 1981). *B. t. taurus* show higher eosinophil counts during secondary infestations compared with *B. t. indicus* breeds in early studies (Tatchell and Moorhouse, 1968). Marufu et al. (2013) confirmed this observation with higher eosinophil counts in tick susceptible Bonsmara cattle, in contrast to other studies which showed higher eosinophil counts in tick resistant cattle (Nelore *B. t. indicus*) in Brazil and Australian Shorthorn breed (*B. t. taurus* tropically adapted cattle) (Schleger et al., 1976; Carvalho et al., 2010b). Using the same breeds as Carvalho et al. (2010b), Franzin et al. (2017) showed higher eosinophil counts in susceptible cattle during the first infestation of tick naïve cattle, yet higher eosinophil counts at nymph feeding sites in the tick resistant breed. Suppression of Th1 responses by eosinophils may be logically associated with the response of resistant cattle and may also correlate with mast cell activity.

Mast cells (including tissue basophils) are a multifunctional cell population involved in maintaining local homeostasis of connective tissue, control of blood coagulation and defensive functions of innate and adaptive immunity. In addition mast cell dysfunction is associated with several chronic allergic/inflammatory disorders (da Silva et al., 2014). Mast cells contain granules rich in histamine and heparin, and are the main effectors of allergic reactions. Host resistance to ticks appears to concur with mast cell functions such as allergic responses, wound healing and immune tolerance, and a potential mast cell dysfunction in tick-susceptible cattle. Most studies on cattle have shown higher mast cell counts in resistant breeds in response to ticks (Table 2). However, one study comparing several breeds found that the Nelore *B. t. indicus* resistant breed had the highest number of mast cells in response to ticks while the Gyr *B. t. indicus* tick resistant breed had similar levels as two tick susceptible *B. t. taurus* breeds, Holstein and Brown Swiss (Veríssimo et al., 2008). In contrast, previously Gyr *B. t. indicus* cattle showed a higher number of mast cells in the dermis compared to susceptible European breeds (Moraes et al., 1992; Veríssimo et al., 2008). A few mast cell activators (da Silva et al., 2014) have been noted in resistant host gene expression studies above including Cathepsins B, D, and L2, platelet activating factors, complement factor C3, IL10, IL2, and TNFα (Table 1).

Basophils are known for their allergic effector function and were first described in response to ticks by Trager in 1939. Basophils notably accumulated at tick bite sites causing cutaneous hypersensitivity (Trager, 1939). In the 1950s, it was confirmed that histamines are stored in basophil granules (Graham et al., 1955). Basophils have been associated with immunity against parasites including ticks and helminths, reviewed by Karasuyama et al. (2011). It is thus logical as described above for mast cells, that high levels of circulating basophils would be associated with the tick resistant phenotype, which has been confirmed by two groups (Carvalho et al., 2010a; Marufu et al., 2014; Franzin et al., 2017). The release of histamine has been postulated as a mechanism which dislodges feeding ticks. This was confirmed when mice were injected with cultured mast cells which resulted in tick rejection following infestation of *Haemophysalis longicornis* ticks, with no tick rejection in mast cell deficient mice (Matsuda et al., 1987). A recent review of basophil functions confirms their effector role in allergic reactions, however basophils also share features of innate and adaptive immunity (Steiner et al., 2016) which again associates well with the tick resistant bovine phenotype. Steiner et al. (2016) examined the ever expanding function of basophils including the modulation of several cytokines, Toll-like receptors and chemokines (including CXCL10, CCR1, CCR7 described as up-regulated in certain tick resistant cattle studies, Table 1).

The correlation of granulocyte activity (and their immune effector mechanisms) in the skin of tick resistant cattle could further be examined to attempt to correlate immunity with gene expression studies described above. The existence of tick histamine-binding salivary lipocalins have been associated with inhibiting histamines from their receptors (Paesen et al., 1999; Mans et al., 2008) with specific lipocalins up-regulated in resistant vs. susceptible breeds in response to ticks (Rodriguez-Valle et al., 2013). In addition, the central role of histamine in tick resistance was supported by antihistamine administration to cattle which led to increased tick loads on both *B. t. taurus* (Hereford) and *B. t. indicus* (Brahman) breeds (Tatchell and Bennett, 1969).

In summary, these studies confirm that cattle breeds behave differently during *R. microplus* infestation, presenting various intrinsic mechanisms to provide protection against ticks. Overall, resistant cattle appear to be associated with increased mast cells, eosinophils, and basophils in the skin, while the recruitment of neutrophils is potentially associated with tick susceptibility. The release of histamines from these cells appears to be associated with the resistant phenotype. Histamine is thought to inhibit tick attachment and leads to itching, which subsequently leads to more grooming and tick removal.

## Microbiota role in tick resistance

The microbiome contributes to the architecture and function of tissues, host energy metabolism, and also plays an important role in the balance between health and disease as demonstrated recently for intracellular protozoa (Yilmaz et al., 2014; Bär et al., 2015). In vertebrates, semiochemicals can be generated by the activity of the microbiota upon amino acids, short chain fatty acids or hormones secreted in body emissions, such as sweat, tears, sebum, saliva, breath, urine, and feces (Amann et al., 2014; Fischer et al., 2015). This volatile repertoire is of paramount importance with evidence that they can direct host-vector specificity (Smallegange et al., 2011; Davis et al., 2013). The variation in the host chemical production thus causes differential attractiveness to vectors between species and in turn, the bacterial profiles differ according to human genetic background (Benson et al., 2010; Prokop-Prigge et al., 2015). Microbial composition can be affected by diet and other management strategies, such as those used for beef and dairy cattle (Durso et al., 2012; Thoetkiattikul et al., 2013). The differences in the composition of their microbiota (Dowd et al., 2008; Durso et al., 2010, 2012; de Oliveira et al., 2013; Mao et al., 2015), as well as the distinct volatile organic compounds (VOC) produced by *B. t. taurus* and *B. t. indicus* cattle may corroborate the contrasting tick infestation phenotypes observed among these animals (Steullet and Gnerin, 1994; Osterkamp et al., 1999; Borges et al., 2015; Ferreira et al., 2015). Although a few studies have demonstrated that host VOCs play a role in attracting *Rhipicephalus* spp. ticks (Louly et al., 2010; Borges et al., 2015; de Oliveira Filho et al., 2016; Franzin et al., 2017), research is still needed to investigate the interrelationship of host microbiota with VOC production related with tick attraction or repulsion. This may potentially reveal yet other factors contributing to tick susceptibility thereby presenting new opportunities to develop control methods for the livestock ectoparasite, *R. microplus*.

## Future research agenda

Understanding the mechanisms behind genetic resistance to ticks and tick-borne diseases in livestock could improve breeding programs to develop cattle that are more resistant and productive (reviewed by Mapholi et al., 2014). Research to identify host genetic markers associated with tick susceptibility or resistance have been limited and compounded by the comparison of local breeds in different geographic regions as summarized here in this review. In addition, several studies reviewed here applied gene expression analysis of tick resistant breeds such as Nelore or Brahman. Brahman cattle have diverged through “up-breeding” in Australia and are thought to contain ~7–10% *B. t. taurus* in their genomes (Bolormaa et al., 2011), whereas the Nelore breed is viewed as highly pure *B. t. indicus* in comparison (<1% *B. t. taurus* content). High throughput genomics is increasingly affordable and thus prior to the identification of tick resistance markers, it would be practical to first determine the genomic differences between and within breeds under study.

Further research assessing tick:host preference mechanisms are still needed. Whether these can be manipulated to protect susceptible cattle from ticks is yet to be determined. The volatile organic compounds of susceptible cattle could be influenced by the microbiome which in turn may be controlled by diet. Recently it was demonstrated that butyric acid (also a VOC, commonly found in feces) stimulates bovine neutrophils and potentiates platelet activating factors thus modulating the innate immune response (Carretta et al., 2016). Indeed, to identify links between tick host attraction and bovine immune responses would be interesting. The potential to manipulate volatiles through probiotic treatments and/or diet could be a future option for tick control.

Within the host immune studies compared in this review, it was apparent that the conditions of the experiments preclude direct comparisons of in particular, gene expression data. Nonetheless some similarities were identified. Future research could focus on proteomic analyses of tick lesions between resistant and susceptible breeds with the recommendation to use tick naïve cattle with multiple skin sampling from early infestations (i.e., initial attachment of larvae) until tick resistance is achieved after several infestations. The studies reported most likely are hampered by the costs associated with long cattle experiments. This could be why many studies held cattle in tick infested pastures during artificial tick infestations as this is the most economical option for long term studies.

Tick vaccines can potentially protect the host from tick infestations and tick borne diseases. This review concentrated less on the development of tick vaccines as it was considered that to develop a successful tick vaccine it would be wise to understand the most common immune effectors to emulate this outcome when using a new anti-tick vaccine candidate(s). Perhaps CRISPR/Cas9 gene editing technologies (parasite CRISPR/Cas9 models recently reviewed by (Cui and Yu, 2016) could be manipulated to examine host-tick relationships in order to demonstrate the most effective tick resistance pathway(s) to be exploited for vaccine development. Indeed CRISPR/Cas9 host editing to favor tick resistance could also be exploited in the future. Further insights into the immunomodulatory processes between ticks and tick susceptible/resistant hosts could identify major genes which would facilitate tick control strategies and the development a broad-spectrum anti-tick vaccine.

## Conclusions

Taking advantage of recent advances from new approaches and technologies as applied to the field of vector biology, such as transcriptomics, proteomics, immune-molecular characterization, elucidation of naturally acquired resistance, and the development of innovative arthropod and animal models, may lead to improved investigations of naturally acquired resistant breeds against tick and tick-borne pathogens. Immune-proteomic, sialotranscriptome and reverse genetics/gene editing (RNAi, CRISPR/Cas9) may help to identify new vaccine candidates that resist ticks and tick-borne pathogens. By understanding the tick:host interface and the most common denominators of immunity to ticks, this acquired immune response could be manipulated to improve the efficacy of novel anti-tick vaccines. Conversely, the knowledge obtained may assist in the selection of tick resistant cattle or the manipulation of susceptible cattle to develop a protective tick immune response. Furthermore, the in depth analysis of host microbiota and volatile organic compounds could lead to probiotic or diet changes or inhibitory chemicals which could render susceptible cattle less attractive to ticks. Future research may lead to a combination of several of these technologies as novel tick and tick-borne disease control options by first identifying viable biological targets and dissecting pathways leading to vaccination or pharmaceutical therapies or cattle management opportunities for tick control.

## Author contributions

AA, GR, and AT conceived the research review. AT prepared the comparative Tables/Figures and with NJ revised all sections written by other co-authors. GRG, AZ, TM and NJ wrote or contributed to specific sections within the review.

### Conflict of interest statement

The authors declare that the research was conducted in the absence of any commercial or financial relationships that could be construed as a potential conflict of interest.
